# Sequence analysis of the *Hex A* gene in Jacob sheep from Bulgaria

**DOI:** 10.14202/vetworld.2021.56-60

**Published:** 2021-01-08

**Authors:** Boyko Neov, Jivko Krastanov, Teodora Angelova, Nadezhda Palova, Stayka Laleva, Peter Hristov

**Affiliations:** 1 Department of Animal Diversity and Resources, Institute of Biodiversity and Ecosystem Research, Bulgarian Academy of Sciences, Sofia 1113, Bulgaria; 2 Department of Breeding and Technologies in Cattle Breeding, Agricultural Institute – Stara Zagora, Agricultural Academy, Stara Zagora 6000, Bulgaria; 3 Scientific Center of Agriculture, Sredets 8300, Agricultural Academy, Bulgaria

**Keywords:** GM2 gangliosides, Hexosaminidase A gene, human replacement therapy, Jacob sheep, Tay–Sachs disease

## Abstract

**Background and Aim:**

Jacob sheep are a rare ancient breed of sheep believed to have originated from the Mediterranean area but which are now kept throughout the world. These sheep have recently attracted medical interest due to the observation of a genetic disorder in the breed that can be used as an animal model of Tay–Sachs disease (TSD). This study aims to detect mutations in the Hexosaminidase A gene in Jacob sheep based on sequence analysis of the 284-bp fragment situated between exon 11 and intron 11 of the gene, a target sequence for site-specific mutation. This is the first study that has investigated Jacob sheep in Bulgaria for gene-specific mutations.

**Materials and Methods:**

A total of 20 blood samples were collected from Jacob sheep from the Rhodope Mountains. DNA was isolated from these samples, and a specific 284-bp fragment was amplified. The amplified products were purified using a polymerase chain reaction purification kit and sequenced in both directions.

**Results:**

Target sequences were successfully amplified from all 20 investigated sheep. Sequence analysis did not show the homozygous, recessive, missense (G-to-C transition) mutation at nucleotide position 1330 (G1330→C) in exon 11, demonstrating that all of these sheep were a normal genotype (wild-type).

**Conclusion:**

Jacob sheep are considered a potentially useful animal model in advancing the understanding of pathogenesis and developing potential therapies for orphan diseases, such as those characterized by mutant GM2 gangliosides. The clinical and biochemical features of the Jacob sheep model of TSD represent well the human classical late-infantile form of this disorder, indicating that the model can serve as a possible new research tool for further study of the pathogenesis and treatment of TSD.

## Introduction

Jacob sheep, also known as Spanish sheep, many-horned sheep, and piebald sheep, is a unique British breed of domestic sheep that has been bred in England for at least 350 years. Both males and females are horned, sporting two, four, or occasionally six horns. The origin of this breed is under debate, but according to one study that used endogenous retrovirus markers to investigate the history of sheep domestication, it is more closely related to old African and Southwest Asian breeds than to other British breeds [1]. About 30 Jacob sheep are kept at one winery (Midalidare Estate, Mogilovo Village, Chirpan) in the central region of Bulgaria mainly as an attraction for visitors both from Bulgaria and abroad. About ten animals from the Midalidare Estate have been used by the Agricultural Institute of Stara Zagora for breeding purposes, primarily for meat.

Many diseases affecting Jacob sheep are of crucial importance to other animals as well, including an unusual form of asymmetric occipital condylar dysplasia [2] and Tay-Sachs disease (TSD), a type of gangliosidosis that also afflicts humans [3,4]. Various gangliosidoses are common in vertebrate species such as dogs [5,6], cats [7,8], swine [9], and humans [10].

One of the most common diseases observed in Jacob sheep is TSD (Hexosaminidase A [Hex A] deficiency; OMIM 272800) [11], an autosomal recessive lysosomal storage neurodegenerative disorder caused by mutations in the *Hex A* gene, which encodes the α-subunit of lysosomal enzyme β-Hexosaminidase α [12,13]. It is characterized by progressive accumulation of GM2 gangliosides (sialic acid-containing glycosphingolipids), which are most abundant in the nervous system [14-16]. Although TSD-afflicted lambs appear normal at birth, the progressive accumulation of GM2 gangliosides in neurons leads to loss of motor function and cognition, developmental regression, dystonia, blindness, seizures, and eventually death [16-18]. β-N-Acetylhexosaminidase (Hex; EC 3.2.1.52) has recently gained attention as this enzyme catalyzes hydrolysis of GM2 gangliosides [19]. β-N-Acetylhexosaminidase consists of three isozymes, Hex A, Hex B, and Hex S, each comprised two subunits. Hex A is a heterodimer composed of both an α and a β subunit, while Hex B is composed of only two β subunits. TSD (B variant) is caused by chain mutations, resulting in a deficiency of Hex A and Hex S activities but maintaining normal Hex B activity [20]. The primary neuronal storage compounds are the GM2 ganglioside and its sialic acid-free derivative [21].

**Figure-1:**
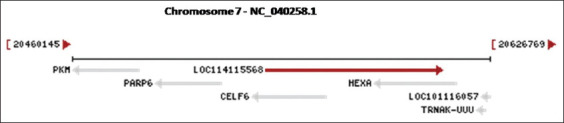
Location of Hexosaminidase A gene on Chromosome 7 (Ovis aries).

The Jacob sheep *Hex A* gene is situated on chromosome 7 (Figure-1) and has a length of 1590 bp, comprising 14 exons and encoding a protein of 529 amino acids [22]. The present study was conducted as research in the literature on mutations in the *Hex A* gene in Jacob sheep is few. This study aimed to detect mutations in the *Hex A* gene in Jacob sheep from Bulgaria.

## Materials and Methods

### Ethical approval

The experiments were carried out under permissions and the guidelines of the Bulgarian Academy of Sciences and the Bulgarian Ministry of Environment and Waters (no. 627/30.03.2015).

### Research period, location, and animals

In Bulgaria, Jacob sheep are represented by a single flock (about 100 animals) located in the Rhodope Mountains. Specimens from this flock have been introduced to the Midalidare Estate winery (Mogilovo village, Central Bulgaria), mainly to attract visitors. Some sheep from Midalidare Estate have been further transferred to the Agricultural Institute of Stara Zagora for breeding. For the purposes of the present study, unrelated animals from the winery and the Agricultural Institute were selected for analysis.

**Figure-2:**
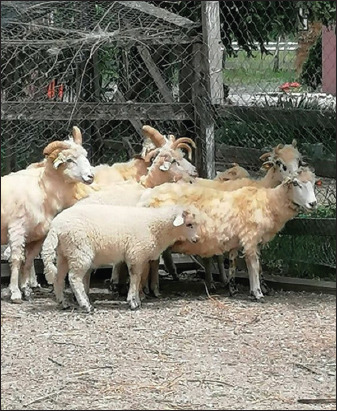
Typical representatives of Jacob sheep from Bulgaria.

The investigation, which took place in 2019, included twenty 4-5-month-old animals – nine males and 11 females (Figure-2). They were typical representatives of the Jacob breed – polycerate, with 4 horns – and did not show neurological symptoms such as ataxia, proprioceptive defects, and cortical blindness. The animals are bred along with the local Stara Zagora sheep breed, but Jacob sheep’s behavior is specific and different, as they are separated and bred independently of the local breed.

### Sample collection and DNA extraction

A total of 20 blood samples (5 mL) from the jugular vein were collected using blood collection tubes (Venoject^®^, Terumo, USA) with K_2_EDTA as an anticoagulant. The samples were obtained from purebred lambs of the Jacob sheep breed provided by the Agricultural Institute of Stara Zagora, Bulgaria.

Total DNA was extracted from whole blood using a GeneMATRIX Tissue DNA Purification Kit (E3550, EURx, Gdansk, Poland), according to the manufacturer’s instructions. DNA concentration was spectrophotometrically determined. The quality of the isolated DNA was verified by 1% agarose gel electrophoresis and then staining with SimpliSafe (E4600-01, EURx, Gdansk, Poland) under UV light. The isolated DNA was stored at −20°C before analysis.

### Polymerase chain reaction (PCR) amplification and sequencing

Based on the sheep genomic reference sequence EU 579459.1 [23], a 284-bp-long fragment between exon 11 and intron 11 from the *Hex A* gene was amplified using the following primers: forward 5′-CTTGCATGAGACTGTGTCCCGGG-3′; reverse 5′-GGGGACCAGGTTTGTGCTGTCC-3′. The primer set covered a partial region of intron 11 (53 bp), the entire exon 11 (186 bp), and a partial sequence of intron 12 (44 bp). All PCR reactions were performed with 10 ng/μl DNA in a final volume of 50 μl (NZYTaq Colorless Master Mix, Cat No. MB040, NZYTech, Portugal). PCR conditions were as follows: Initial denaturation at 94°C for 5 min, 30 cycles of denaturation at 94°C for 30 s, primer hybridization at 50°C for 30 s, elongation at 72°C for 1 min, and final elongation at 72°C for 10 min. In addition, a negative control was also included for all PCR reactions.The amplified fragments were separated and visualized by 1% agarose gel electrophoresis, followed by staining with SimpliSafe (E4600-01, EURx, Gdansk, Poland). Fragment size was determined using the Gene-Ruler™ 100 bp Ladder Plus (Cat. No. SM0323; Thermo Fisher Scientific Inc., Waltham, MA, USA). Amplified products were purified with a PCR purification kit (Gene Matrix, PCR clean-up kit, EURx, Gdansk, Poland) to remove excess primers and nucleotides and were then sequenced in both directions using a PlateSeq Kit (Eurofins Genomics Ebersberg, Germany) per the manufacturer’s instructions.

### Data processing and analysis

All 20 obtained DNA sequences were manually edited and aligned with the MEGA7 software program [24] using the sheep reference DNA sequence EU 579459.1 [23]. Polymorphisms in the gene coding region (the last nucleotide of exon 11 of the *Hex A* gene) in the amplified products were analyzed using DNAStar software (DNAStar Inc., Madison, WI, USA) [25]. The obtained sequences were deposited in GenBank under accession number MT994365.

## Results and Discussion

Jacob sheep were originally called piebald, spotted, or Spanish sheep, and it has been suggested that Jacob sheep were present in England as early as the 1500s [2]. In 2011, a group of researchers published a report showing the results of biochemical and molecular genetic analyses of four Jacob lambs diagnosed with TSD [11].

**Figure-3:**
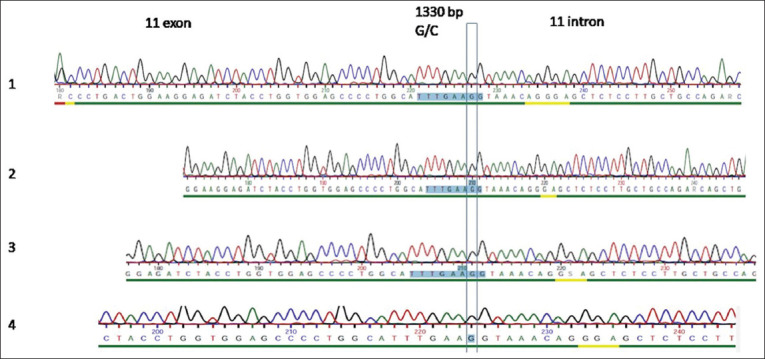
Partial sequence of sheep β-Hex A genomic DNA sequence. There is no mutation at nucleotide position 1330 (G1330→C) in exon 11.

Our results from the partial sequence analysis of the *Hex A* gene did not demonstrate a homozygous, recessive, and missense (G-to-C transition) mutation at nucleotide position 1330 (G1330→C) in exon 11. A BLAST search in the GenBank database found only two additional identical sequences of mRNA, both coding for the *Ovis aries* hexosaminidase subunit alpha protein XM_027971423 and EU579459.1. These sequences covered exon 11 of our sequence entries. Figure-3 clearly shows the absence of even heterozygous animals, that is, expression of both the wild-type and mutant allele of the gene. Thus, our results did not demonstrate this mutation at nucleotide position 1330, and all lambs were determined to be carrying a normal wild-type genotype.

This mutation is uncommon in Jacob sheep. Using a PCR-restriction fragment length polymorphism analysis, investigation of 104 genomic DNA samples from Jacob sheep in three geographically distinct flocks showed the overall incidence of carriers to be only 14.4% (range, 12.5%-18.5%) [26]. The small number of lambs included in our study due to the small number of representatives of this breed in Bulgaria could be a possible reason for not detecting this specific mutation.

Moreover, the sequence analysis from this study did not even find carrier Jacob sheep, that is, animals heterozygous for the wild-type and recessive mutant allele of the gene. Carrier identification is important, because carriers appear normal and live a normal life; however, breeding a mutation-carrying Jacob sheep to a normal Jacob sheep results in a 50% chance of producing another carrier lamb and a 25% chance of a TSD-afflicted Jacob sheep.

One important feature of TSD is that its clinical manifestations in Jacob sheep are the closest to the pathological effects observed in humans [27]. Most importantly, Jacob sheep share a remarkable 86% *Hex A* sequence similarity with humans. Thus, in terms of research, the Jacob sheep are one of the most useful animal models of spontaneously developing TSD. Because of this genetic similarity, the clinical and biochemical features of the Jacob sheep model are likely to mirror well those of the human infantile form of TSD. Furthermore, the level of residual enzyme activity is also similar [26]. This means that treatments such as gene therapy that has been successfully carried out in Jacob sheep may be more likely to succeed in afflicted humans.

## Conclusion

The sequence analysis of the fragment of *Hex A* did not show the specific G1330→C mutation at the 3’-end of exon 11 in any of the investigated lambs; therefore, the studied animals were all normal, possessing the wild-type allele.

This study, the first report examining the genotype of Jacob sheep in Bulgaria, may be used as a starting point for genotyping additional flocks. This will allow the prevention of TSD in sheep through identification of afflicted individuals and will help avoid the breeding of carriers in Jacob flocks.

## Authors’ Contributions

PH and JK conceived and designed the research. BN and TA carried out all experiments and performed the data analysis. NP and SL drafted the manuscript. All authors read and approved the final manuscript.
